# Revised trauma scoring system to predict in-hospital mortality in the emergency department: Glasgow Coma Scale, Age, and Systolic Blood Pressure score

**DOI:** 10.1186/cc10348

**Published:** 2011-08-10

**Authors:** Yutaka Kondo, Toshikazu Abe, Kiyotaka Kohshi, Yasuharu Tokuda, E Francis Cook, Ichiro Kukita

**Affiliations:** 1 Department of Emergency Medicine, Graduate School of Medicine, University of the Ryukyus, 207 Uehara, Nishihara, Okinawa 903-0215, Japan; 2 Department of Emergency Medicine, Mito Kyodo General Hospital, University of Tukuba, 3-2-7, Miyamachi, Mito City, Ibaraki 310-0015, Japan; 3 Emergency Unit, University Hospital of the Ryukyus, 207 Uehara, Nishihara, Okinawa 903-0215, Japan; 4 Institute of Clinical Medicine, Graduate School of Comprehensive Human Sciences, University of Tsukuba, 3-2-7, Miyamachi, Mito City, Ibaraki 310-0015, Japan; 5 Department of Epidemiology, Harvard School of Public Health, 677 Huntington Avenue, Boston, MA 02115, USA

**Keywords:** wounds and injuries, trauma, research design, databases, factual, hospital mortality, scoring system

## Abstract

**Introduction:**

Our aim in this study was to assess whether the new Glasgow Coma Scale, Age, and Systolic Blood Pressure (GAP) scoring system, which is a modification of the Mechanism, Glasgow Coma Scale, Age, and Arterial Pressure (MGAP) scoring system, better predicts in-hospital mortality and can be applied more easily than previous trauma scores among trauma patients in the emergency department (ED).

**Methods:**

This multicenter, prospective, observational study was conducted to analyze readily available variables in the ED, which are associated with mortality rates among trauma patients. The data used in this study were derived from the Japan Trauma Data Bank (JTDB), which consists of 114 major emergency hospitals in Japan. A total of 35,732 trauma patients in the JTDB from 2004 to 2009 who were 15 years of age or older were eligible for inclusion in the study. Of these patients, 27,154 (76%) with complete sets of important data (patient age, Glasgow Coma Scale (GCS) score, systolic blood pressure (SBP), respiratory rate and Injury Severity Score (ISS)) were included in our analysis. We calculated weight for the predictors of the GAP scores on the basis of the records of 13,463 trauma patients in a derivation data set determined by using logistic regression. Scores derived from four existing scoring systems (Revised Trauma Score, Triage Revised Trauma Score, Trauma and Injury Severity Score and MGAP score) were calibrated using logistic regression models that fit in the derivation set. The GAP scoring system was compared to the calibrated scoring systems with data from a total of 13,691 patients in a validation data set using c-statistics and reclassification tables with three defined risk groups based on a previous publication: low risk (mortality < 5%), intermediate risk, and high risk (mortality > 50%).

**Results:**

Calculated GAP scores involved GCS score (from three to fifteen points), patient age < 60 years (three points) and SBP (> 120 mmHg, six points; 60 to 120 mmHg, four points). The c-statistics for the GAP scores (0.933 for long-term mortality and 0.965 for short-term mortality) were better than or comparable to the trauma scores calculated using other scales. Compared with existing instruments, our reclassification tables show that the GAP scoring system reclassified all patients except one in the correct direction. In most cases, the observed incidence of death in patients who were reclassified matched what would have been predicted by the GAP scoring system.

**Conclusions:**

The GAP scoring system can predict in-hospital mortality more accurately than the previously developed trauma scoring systems.

## Introduction

Trauma is a time-sensitive condition. Especially during the first hour of trauma management, assessment, resuscitation and definitive care are very important. Providing definitive care earlier at trauma centers has been shown to decrease mortality [1,2]. Easy-to-use trauma scoring systems inform physicians of the severity of trauma in patients and help them decide the course of trauma management. The use of trauma scoring systems is appropriate in two situations that occur in trauma patient care. They can be used in the field, before the patient reaches the hospital, to decide whether to send the patient to a trauma center. They can also be used for clinical decision making when the trauma patient has just arrived at the emergency department (ED). When the patient is in the ED, trauma scoring systems can be used to prepare the patient for surgery, to call on medical staff for trauma support and to inform the family of the severity of the patient's condition in the early stage.

Many trauma scoring systems have been developed and used. For instance, the Revised Trauma Score (RTS) [3] is most widely cited and used. It also comprises the content of the Trauma and Injury Severity Score (TRISS) [4]. However, calculation of the RTS is too complicated for easy use in the ED. Also, it might not have high reliability when used by paramedics. Moreover, respiratory rate (RR), a component of the RTS, is less reliable than other factors because it is influenced by patient age, mechanism of injury and mechanical ventilation. The Triage RTS (T-RTS) is based on the same risk intervals and variables of the RTS and is simpler to use [3]. However, the T-RTS has the same problems as the RTS. TRISS is also widely used at trauma centers. It strongly predicts probability of survival because it involves the mechanism of the injury as well as anatomical and physiological factors [5], but it is very complex to use.

In addition, the three scoring systems described above may be somewhat dated because trauma situations may have changed since they were developed. Two scoring systems used in the German Trauma Registry have been developed and published [6,7]. They seem more reliable than the previous trauma scoring systems. However, they require laboratory data for scoring, and thus a significant amount of time would be required to obtain results, which might not be immediately available to small hospitals. Moreover, Sartorius *et al*. [5] developed the Mechanism, Glasgow Coma Scale, Age, and Arterial Pressure (MGAP) score as an improvement over the previous simple trauma scores. Thus, it is one of the best and newest scoring systems for predicting in-hospital mortality for trauma patients. However, it also has problems. Its mechanism score is doubtful because it gives higher scores for penetrating trauma, which is not always more severe than blunt trauma. Moreover, the mechanism score based on penetrating trauma usually affects fewer than 10% of all of trauma patients [5,8,9]. Since MGAP is somewhat difficult to use in the clinical setting, we modified the MGAP to create the new Glasgow Coma Scale, Age, and Systolic Blood Pressure (GAP) scoring system. Our purpose was to assess whether the new GAP scoring system better predicts in-hospital mortality among trauma patients than the RTS, T-RTS, TRISS and MGAP scores.

## Materials and methods

### Study design and data collection

This multicenter, prospective, observational study was devoted to the analysis of readily available variables associated with mortality in trauma patients. The data used in this study were derived from the Japan Trauma Data Bank (JTDB), which was established in 2003, with the Japanese Association for the Surgery of Trauma (Trauma Registry Committee) and the Japanese Association for Acute Medicine (Committee for Clinical Care Evaluation) as the main parties. The aim of establishing the JTDB was to collect and analyze trauma patient data in Japan (patient and injury characteristics, information from emergency services, vital signs before reaching the hospital and at the first medical examination, inspections and treatments, diagnosis and Injury Severity Score (ISS) [10,11], disposition after being in the ED or the operating room (OR) and information upon discharge from the hospital). The severity of anatomic injuries was evaluated using the ISS. Trauma scores such as the RTS, the T-RTS and the MGAP were calculated using these data. Probability of survival was also calculated on the basis of the TRISS [4].

114 major emergency hospitals in Japan contributed data to the JTDB registry. Among 221 certified tertiary level emergency medical centers, 93 institutions (42%) participated. These hospitals have ability equivalent to level I trauma centers in the United States. Data were collected from participating institutions via the Internet [8]. In most cases, the physicians who attended the Abbreviated Injury Scale (AIS) [12] coding course registered the data. The AIS score, a component of the ISS, was recorded using the AIS 90 Update 98 [13].

We received permission to use the data from the JTDB. The ethics committee at our institution does not require its approval for observational studies using anonymous data such as those used in this study.

### Selection of participants

**Figure 1:**
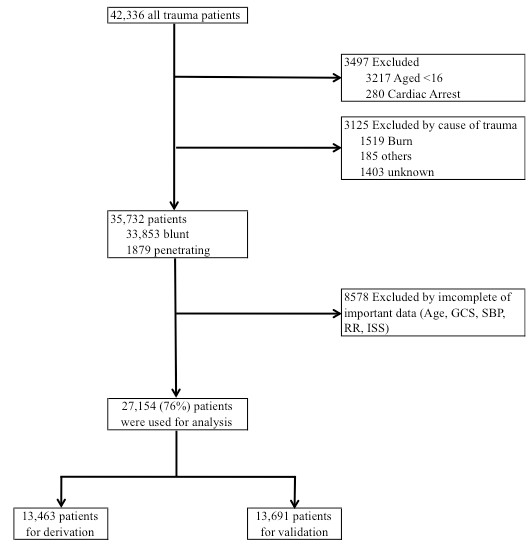
**Schematic showing the patient selection procedure**. GCS: Glasgow Coma Scale; SBP: systolic blood pressure; RR: respiratory rate; ISS: Injury Severity Score.

A total of 42,336 patients in this study were enrolled in the JTDB from 2004 to 2009. Our study included trauma patients from the JTDB who had ISS > 3. A total of 6,622 patients were excluded because they were 15 years of age or younger, had died during the initial examination at the trauma scene or other trauma mechanism such as burn. Thus, 35,732 patients met our study criteria (Figure 1). Among those patients, we used data from 27,154 patients (76%) for analysis with complete data sets on important parameters (patient age, GCS score, SBP, RR and ISS) (Figure 1).

### Statistical analysis

Patient data displayed are means ± standard deviations (SDs) or raw numbers followed by percentages in parentheses. The primary end point was death at discharge. The secondary end point was death in the ED or the OR.

First, we randomly divided this cohort into two parts: a derivation data set and a validation data set. Of 27,154 patients, 13,463 were assigned to the derivation data set and 13,691 were assigned to the validation data set (Figure 1). Second, we reconsidered the MGAP score by conducting a clinical review to create the GAP. Construction of the thresholds for each predictor in the MGAP was based on clinical knowledge. The points applied to each category were determined by the relative sizes of the regression coefficients from a logistic regression model that was fit to the derivation data set to predict mortality. Thus, we left in GCS score, patient age and SBP as predictors in our GAP scoring system. Since penetrating trauma seldom occurs (< 10%) and does not include other clinically important features, such as the location of the injury, we decided to exclude this predictor from our scale. The trauma mechanism should be used with an anatomical score such as the TRISS. Comparison of each trauma score on the validation data set was assessed by c-statistics. Finally, to compare the GAP score with trauma scores such as MGAP, RTS, T-RTS and TRISS, we calibrated each score to our population by fitting a logistic regression model to predict the probability of mortality in the derivation data set. The predicted mortality probabilities from these models were then collapsed into three categories previously described by Sartorius *et al*. [5]: trauma patients at low risk (< 5%), intermediate risk, and high risk (> 50%) of death.

Reclassification tables were constructed [14] to compare the GAP scoring system to each of the existing scoring systems on the validation data set. We also performed subgroup analysis of the validation data set. The subgroup was patients with an ISS > 16, who were classified as severe trauma patients. Comparison of the accuracy of the GAP score, which depended on trauma severity in the validation data set, was also assessed by c-statistics. All analyses were performed with SAS version 9.2 statistical software (SAS Institute, Cary, NC, USA).

## Results

The main characteristics of the trauma patients in each cohort are shown in Table 1. The patients' mean age (± SD) was 51.2 ± 21.4 years, and 68.9% of the patients were men. Almost all blunt trauma patients (94.6%) and 3,302 penetrating trauma patients (12.2%) had been drinking alcohol prior to the developed trauma. These results represent combined derivation and validation data. Table 2 displays the characteristics of treatments and the outcomes of the trauma patients in each cohort. Prehospital treatment is simple in Japan. Overall mortality at discharge in our cohort was 15.0% (3,270 of 21,788 patients) and the mortality rate in the ED or OR was 5.4% (1,327 of 24,756 patients). These results are also combined derivation and validation data.

**Table 1:** Characteristics of trauma patients^a^

				Derivation	Validation
Characteristics		Statistical measurement	Units	(*N *= 13,463)	(*N *= 13,691)
Patient age		Means ± SD	Years	51.2 ± 21.3	51.2 ± 21.5
Gender (male)		*n *(%)		9,270 (68.9)	9,434 (68.9)
Trauma causes	Blunt	*n *(%)		12,742 (94.6)	12,939 (94.5)
	Penetrating	*n *(%)		721 (5.4)	752 (5.5)
	Ambulance	*n *(%)		11,285 (87.2)	11,511 (87.2)
	Helicopter	*n *(%)		849 (6.6)	841 (6.4)
Transporter	Doctor's car	*n *(%)		405 (3.1)	422 (3.2)
	Own car	*n *(%)		244 (1.9)	250 (1.9)
	On foot	*n *(%)		64 (0.5)	62 (0.5)
	Other	*n *(%)		99 (0.8)	109 (0.8)
	SBP	Means ± SD	mmHg	131.9 ± 31.0	132.5 ± 31.0
	DBP	Means ± SD	mmHg	77.4 ± 20.0	77.4 ± 20.2
Prehospital vital sign	HR	Means ± SD	per minute	85.3 ± 23.4	85.0 ± 23.5
	RR	Means ± SD	per minute	22.2 ± 7.4	22.0 ± 7.4
Alcohol drunk		*n *(%)		1,677 (12.5)	1,625 (11.9)
	GCS	Means ± SD		12.6 ± 4.0	12.6 ± 4.0
	SBP	Means ± SD	mmHg	125.3 ± 44.7	125.3 ± 44.7
	DBP	Means ± SD	mmHg	74.5 ± 24.0	74.3 ± 24.4
Initial vital signs at ED	HR	Means ± SD	per minute	82.4 ± 28.4	82.3 ± 28.9
	RR	Means ± SD	per minute	20.8 ± 8.5	20.8 ± 8.6
	BT	Means ± SD	°C	36.2 ± 1.0	36.3 ± 1.0
	ISS	Means ± SD		16.9 ± 13.5	17.1 ± 13.5
	RTS	Means ± SD		6.9 ± 2.0	6.8 ± 2.0
	T-RTS	Means ± SD		10.6 ± 2.9	10.6 ± 3.0
Trauma scores	TRISS	Means ± SD		0.85 ± 0.28	0.84 ± 0.28
	MGAP	Means ± SD		23.5 ± 5.5	23.4 ± 5.6
	GAP	Means ± SD		19.4 ± 5.2	19.3 ± 5.3

^a^Missing prehospital data are gender (*n *= 7), transporter (*n *= 1,009), SBP (*n *= 7,364), DBP (*n *= 10,131), HR (*n *= 5,797), RR (*n *= 7,386). Missing data at the ED are alcohol drunk (*n *= 9,649) and BT (*n *= 3,542). SBP: systolic blood pressure; DBP: diastolic blood pressure; HR: heart rate; RR: respiratory rate; GCS: Glasgow Coma Scale; BT: body temperature; ISS: Injury Severity Scale; RTS: Revised Trauma Score; T-RTS: Triage-RTS; TRISS: Trauma and Injury Severity Score; MGAP: Mechanism, Glasgow Coma Scale, Age, and Arterial Pressure; GAP: Glasgow Coma Scale, Age, and Systolic Blood Pressure; ED: emergency department; SD: standard deviation.

**Table 2:** Characteristics of treatment and outcomes for trauma patients^a^

		Derivation, *n *(%)	Validation, *n *(%)
Treatments and diagnostic tools		(*N *= 13,463)	(*N *= 13,691)
	O_2_	7,928 (59.0)	8,097 (59.1)
	Cervical collar	6,932 (51.5)	7,092 (51.8)
Prehospital treatment	Backboard	6,257 (46.5)	6,469 (47.3)
	Shock pants	9 (0.1)	12 (0.1)
	Intravenous fluid	547 (4.1)	496 (3.6)
	Positive	1,128 (9.0)	1,211 (9.5)
FAST	Negative	8,884 (71.1)	8,974 (65.5)
	Not used	2,475 (19.8)	2,554 (20.0)
	Head	8,901 (66.1)	9,130 (66.7)
	Neck	4,506 (33.5)	4,570 (33.4)
	Spine	1,591 (11.8)	1,629 (11.9)
CT	Chest	6,563 (48.7)	6,691 (48.9)
	Abdomen	6,463 (48.0)	6,564 (46.9)
	Pelvis	5,099 (37.9)	5,192 (37.9)
	Others	504 (3.7)	471 (3.4)
	Head	129 (1.0)	107 (0.8)
	Neck	52 (0.4)	62 (0.5)
	Spine	21 (0.2)	29 (0.2)
Angiography	Chest	109 (0.8)	121 (0.9)
	Abdomen	377 (2.8)	389 (2.8)
	Pelvis	424 (3.1)	428 (3.1)
	Others	52 (0.4)	44 (0.3)
Blood transfusion (+)		2,166 (17.2)	2,216 (17.2)
	Craniotomy	470 (3.5)	463 (3.4)
	Craterization	174 (1.3)	151 (1.1)
	Thoracotomy	263 (2.0)	275 (4.2)
Operation	Celiotomy	545 (4.0)	541 (4.0)
	Bone Fixation	2,170 (16.1)	2,117 (15.5)
	Angiostomy	88 (0.7)	77 (0.6)
	TAE	383 (2.8)	404 (3.0)
	Endoscopic surgery	7 (0.1)	14 (0.1)
	Anastomosis	48 (0.4)	45 (0.3)
	Others	438 (3.3)	487 (3.6)
	Death in ED or OR	655 (5.3)	672 (5.4)
Disposition at ED	ICU	8,464 (68.9)	8,701 (69.7)
	Ward	2,904 (23.7)	2,826 (22.6)
	Others	254 (2.1)	280 (2.2)
	Death	1,600 (14.8)	1,670 (15.2)
Disposition at discharge	Hospital transfer	4,350 (40.2)	4,375 (40.0)
	Home	4,811 (44.4)	4,864 (44.4)
	Others	63 (0.6)	55 (0.5)

^a^Missing data are FAST (*n *= 1,928), blood transfusion (*n *= 1,683), disposition at ED (*n *= 2,398), and disposition at discharge (*n *= 5,366). FAST: Focused Assessment with Sonography for Trauma; CT: computed tomography; TAE: transcatheter arterial embolization; ED: emergency department, OR: operation room.

Table 3 displays the results of the logistic regression used to develop the GAP scoring system using the derivation cohort. The predictors were categorized in the same way that Sartorius *et al*. [5] described when they developed the MGAP. The GCS score was entered into the model without any modification. SBP was segmented into three categories (< 60 mmHg, 60 to 120 mmHg and > 120 mmHg). Patient age was dichotomized into two categories (< 60 years or ≥ 60 years). The β coefficients (standard errors (SE)) were -0.35 (0.110 for the initial GCS score at the ED) and -1.01 (0.080 for the younger age group (< 60 years). For SBP, the β coefficients (SEs) were -1.93 (0.11) for SBP > 120 mmHg and -1.23 (0.12) for patient age 60 years ≤ SBP ≤ 120, respectively. The point system used to calculate the GAP scores was the same as that used to calculate the MGAP scores: the relative size of the β coefficients. The points used to calculate the GAP scores were GCS score (from three to fifteen points), patient age (< 60 years, three points) and SBP (> 120 mmHg, six points; 60 to 120 mmHg, four points).

**Table 3:** Multivariate analysis of predictors of in-hospital death to develop the GAP in the derivation data set^a^

Parameter	Values	β coefficient (SE)	GAP score
Initial GCS at ED	GCS value	-0.35 (0.11)	3 to 15
Patient age	< 60 years	-1.01 (0.08)	3
	> 120	-1.93 (0.11)	6
Initial systolic blood pressure at ED	60 to 120	-1.23 (0.12)	4
	< 60	Reference	0

^a^GAP: Glasgow Coma Scale; Age, and Systolic Blood Pressure; ED: emergency department; GCS: Glasgow Coma Scale; SE: standard error.

The c-statistics of the GAP scores in the validation data set (0.933 for long-term mortality and 0.965 for short-term mortality) were better than or comparable to those of the MGAP score (0.924 and 0.954, respectively), the RTS (0.919 and 0.966, respectively) and the T-RTS (0.917 and 0.969, respectively), but slightly less than that of the TRISS (0.948 and 0.969, respectively) (Table 4).

**Table 4:** C-statistics of performance of RTS, T-RTS, TRISS, MGAP and GAP in the validation data set^a^

Scoring system	Long-term mortality	Short-term mortality
RTS	0.919	0.966
T-RTS	0.917	0.968
TRISS	0.948	0.969
MGAP	0.924	0.954
GAP	0.933	0.965

^a^RTS: Revised Trauma Score; T-RTS: Triage RTS; TRISS: Trauma and Injury Severity Score; MGAP: Mechanism, Glasgow Coma Scale, Age, and Arterial Pressure; GAP: Glasgow Coma Scale, Age and Systolic Blood Pressure.

We divided the population into three risk categories to illustrate the incidence of death clearly: trauma patients at low risk (< 5%), intermediate risk, and high risk (> 50%) of death. The range of the GAP scores was determined for each risk category to match the range of predicted risk of death using the derivation data set: severe (high risk: 3 to 10 points), moderate (intermediate risk: 11 to 18 points) and mild (low risk: 19 to 24 points). A total of 1,409 (10.3%) of the patients were assigned to the high-risk group and had an observed mortality rate of 74.2%. A total of 2,044 (14.9%) of the patients were assigned to the intermediate-risk group and had an observed mortality rate of 21.4%. A total of 10,238 (74.7%) of the patients were assigned to the mild-risk group and had an observed mortality rate of 1.8%. The MGAP classified patients as severe (high risk: 3 to 17 points), moderate (intermediate risk: 18 22 points) and mild (low risk: 23 to 29 points) in their study [5]. However, the MGAP was recalibrated as follows: severe (high risk: 3 to 14 points), moderate (intermediate risk: 15 to 22 points) and mild (low risk: 23 to 29 points), according to the predicted risk of death in the derivation data set determined by logistic regression. The other scores were recalibrated using logistic regression as well. Table 5 shows the reclassification of trauma severity using the validation data set on the basis of previously used trauma scoring systems and the GAP scoring system. All cases except one moved in the correct direction on the basis of the GAP score. For example, the RTS classified 9,654 patients as having mild trauma (predicted mortality risk < 5%). However, the GAP identified a subset of 205 of these patients as having moderate trauma severity with a mortality rate of 10.2%, which is within the range of intermediate mortality risk (5% to 50%). In addition, the RTS classified 2,985 patients as having moderate trauma, but the GAP correctly reclassified 367 of these patients as having severe trauma (mortality rate 55.6%, matching predicted risk > 50%). Moreover, the GAP scoring system reclassified another 789 patients as having mild trauma. Although the mortality rate of these patients (7.4%) was greater than that predicted for low-risk patients (< 5%), the observed mortality rate was much less than that of the 1,829 patients who were not reclassified (22.5%). Finally, the RTS classified 1,052 patients as having severe trauma, but the GAP scoring system reclassified 10 of these patients as having moderate trauma. Although the mortality rate of the reclassified patients (60.0%) was greater than the predicted range for intermediate-risk patients (5% to 50%), the mortality rate of these patients was less than that of patients who were reclassified (80.8%). In summary, all patients who were reclassified according to the GAP scoring system were correctly moved into categories of higher or lower risk compared to their initial risk according to the RTS. Likewise, the mortality rates of 2,581, 2,141 and 1,384 patients also moved in the correct direction when the GAP scoring system was compared with the T-RTS, TRISS and MGAP, respectively. In most cases, the observed incidence of death in patients who were moved to different risk categories by the GAP scoring system matched what would have been predicted by the GAP system.

**Table 5:** Reclassification of severity between the previous trauma scores and the GAP in the validation data set^a^

Reclassification comparisons					
Reclassification of severity between RTS and GAP					
		RTS			
Scoring system	Severity	Severe (< 3.4 points)	Moderate (3.4 to 7.2 points)	Mild (> 7.2 points)	Total
	Severe (3 to 10 points)	1,042 (80.8)	367 (55.6)	0	1,409 (74.2)
GAP	Moderate (11 to 18 points)	10 (60.0)	1,829 (22.5)	205 (10.2)	2,044 (14.9)
	Mild (19 to 24 points)	0	789 (7.4)	9,449 (1.4)	10,238 (1.8)
	Total	1,052 (80.6)	2985 (22.6)	9,654 (1.5)	13,691 (12.2)
Reclassification of severity between the TRTS and the GAP					
		TRTS			
Scoring system	Severity	Severe (< 6 points)	Moderate (6 to 11 points)	Mild (> 11 points)	Total
	Severe (3 to 10 points)	968 (82.3)	441 (56.5)	0	1,409 (74.2)
GAP	Moderate (11 to 18 points)	10 (60.0)	1,869 (22.4)	165 (7.9)	2,044 (14.9)
	Mild (19 to 24 points)	0	1,965 (4.6)	8,273 (1.2)	10,238 (1.8)
	Total	978 (82.1)	4,275 (17.7)	8,438 (1.3)	13,691 (12.2)
Reclassification of severity between TRISS and GAP					
		TRISS			
Scoring system	Severity	Severe (< 0.236 point)	Moderate (0.236 to 0.935 point)	Mild (> 0.935 point)	Total
	Severe (3 to 10 points)	1,124 (79.4)	284 (54.2)	1 (0)	1,409 (74.2)
GAP	Moderate (11 to 18 points)	90 (58.9)	1,560 (23.8)	394 (3.6)	2,044 (14.9)
	Mild (19 to 24 points)	11 (45.4)	1362 (8.2)	8,865 (0.8)	10,238 (1.8)
	Total	1,225 (77.6)	3,206 (19.9)	9,260 (0.9)	13,691 (12.2)
Reclassification table of severity between MGAP and GAP					
		MGAP			
Scoring system	Severity	Severe (3 to 14 points)	Moderate (15 to 22 points)	Mild (23 to 29 points)	Total
	Severe (3 to 10 points)	1,287 (75.3)	122 (63.1)	0	1,409 (74.2)
GAP	Moderate (11 to 18 points)	83 (32.5)	1,828 (21.5)	133 (13.5)	2,044 (14.9)
	Mild (19 to 24 points)	0	1,046 (5.3)	9192 (1.4)	10,238 (1.8)
	Total	1,370 (72.7)	2,996 (17.5)	9,325 (1.6)	13,691 (12.2)

^a^Column data are number of deaths (%). Severe: high risk (> 50%) of death; Moderate: intermediate risk of death; Mild: low risk (< 5%) of death. RTS: Revised Trauma Score; T-RTS: Triage RTS; TRISS: Trauma and Injury Severity Score; MGAP: Mechanism, Glasgow Coma Scale, Age and Arterial Pressure; GAP: Glasgow Coma Scale, Age, and Systolic Blood Pressure.

In subgroup analysis, 6,552 patients had severe trauma (ISS > 16) in the validation data set (*n *= 13,691). The c-statistic for the GAP scoring system in severe trauma patients (0.905 for long-term mortality and 0.943 for short-term mortality) was comparable to the c-statistics for the GAP scoring system in all trauma patients in the validation data set.

## Discussion

The goal of our study was to modify the MGAP, which is one of the best trauma scoring systems to be applied easily in the ED. We found that the GAP score is a better predictor and more generalizable than the MGAP score. The c-statistics given in Table 4 show that the GAP score predicts trauma severity as well as or better than the other trauma scores, including the MGAP. Also, the GAP score is easier to calculate than the others. The reclassification table (Table 5) shows the improved prediction value of the GAP score over the trauma scores. In Table 5, the trauma severity of all patients except one moved in the correct direction on the basis of the GAP score, although some groups' mortality rates were not as low as those predicted by the categories. However, almost all of the mortality rates for each category of the GAP scoring system were compatible with those predicted by the GAP.

Presumably, eliminating the trauma mechanism score from the MGAP would result in some misclassification. Some studies have shown that penetrating trauma is more severe than blunt trauma [4]. However, these scores should reflect both where and how patients sustained trauma, such as the TRISS, when physicians see trauma patients. The trauma mechanism score might not work without an anatomical score. Moreover, penetrating trauma patients have been found to comprise fewer than 10% of the trauma patient population in other countries [5,9], although there are few trauma patients with gunshot wounds in Japan.

The results of TRISS showed slightly better than the results of the GAP. However, the TRISS is not as useful as the GAP in the early stages of trauma treatment in the ED, because it requires information not readily available at the time of presentation to the ED. Although the TRISS may better predict survival than the GAP score, the TRISS also involves some calculations that may limit its use. The GAP scoring system is easier to use than TRISS and provides valuable predictive information to the ED. Therefore, from that viewpoint, we think that it is one of the best trauma prediction scoring systems.

By using the GAP scoring system, we identified 10.3% and 74.7% of patients who were at high and low risk of death, respectively. Both percentages were the highest among the trauma scoring systems. This suggests that the GAP scoring system is the more applicable to the clinical setting. Moreover, if we set trauma severity as low risk (< 10%), intermediate risk, and high risk (> 50%) of death, the GAP scoring system more accurately predicts mortality risk categories.

Many trauma studies, including those evaluating that these studies' researchers first published the RTS, T-RTS, TRISS and MGAP, have been based on prehospital data. However, generally speaking, they are now more widely used in the ED than in the prehospital setting. If these scoring systems are used in the ED, the scores should be assigned on the basis of data derived in the ED. Actually, the MGAP is a prediction rule that is targeted more for use in the ED than in the prehospital setting because its data come from mobile ICUs. Since mobile ICUs start treatment in the ambulance similarly to treatment in the ED, their prediction model should deal with ED prediction.

This study has a few limitations. First, because of missing data among some patients, we used only 76% of the eligible patients for analysis. Thus the study might have a selection bias. However, more than 25% of the patients in the American College of Surgeons National Trauma Data Bank are also missing physiological information [15]. However, most previous trauma studies have had more missing data than ours [16]. Although Moore *et al*. [17] recommended using multiple imputation as a more accurate data model, we chose the simple approach of eliminating all patients with missing data because of the size of our data set. Since our final scoring system was based on the relative size of the β coefficients, it may not have been influenced by small changes in β coefficients from imputation. Second, the β coefficients for the GAP scoring system were derived from fitting a logistic regression model to our derivation data set. The weights for the predictors in the other scoring systems were based on other data sets. Those scoring systems were recalibrated in our derivation data set. Moreover, all comparisons of scoring systems were performed using a separate validation data set. However, we need to evaluate the performance of the GAP scoring system in other populations to ensure external validity. Third, the JTDB data have a potential selection bias because hospitals that participate in the JTDB do so voluntarily and the registered records were not consecutive [8]. Nevertheless, our data and the data reported in previous trauma studies probably do not have major differences with regard to the characteristics of patients. Thus, generalization of our study results may not be a problem.

## Conclusions

Our new simple trauma scoring system, the GAP scoring system, strongly predicts in-hospital mortality. It will lead to improved survival of trauma patients and provide physicians with future decision-making schemes.

## Key messages

• We have developed a simple, new trauma scoring system: the GAP scoring system. The components of the GAP score are the GCS score (from three to fifteen points), patient age (< 60 years, three points) and SBP (> 20 mmHg, six points; 60 to 120 mmHg, four points).

• The GAP score is simpler, more generalizable and a better predictor of in-hospital mortality than previous trauma scale scores.

## Abbreviations

AIS: Abbreviated Injury Scale; GAP: Glasgow Coma Scale, Age, and Systolic Blood Pressure; GCS: Glasgow Coma Scale; ED: emergency department; ISS: Injury Severity Score; JTDB: Japan Trauma Data Bank; MGAP: Mechanism, Glasgow Coma Scale, Age, and Arterial Pressure; OR: operation room, RR: respiratory rate; RTS: Revised Trauma Score; SBP: systolic blood pressure; SD: standard deviation; SE: standard error; TRISS: Trauma and Injury Severity Score.

## Competing interests

The authors declare that they have no competing interests.

## Authors' contributions

YK, KK and IK contributed to the acquisition of data. YK, TA and EFC jointly conceived of and designed this study. TA conducted data cleaning. TA and EFC jointly analyzed and interpreted the data. TA drafted the manuscript. All of the authors reviewed and discussed the manuscript. TA, YT and EFC revised the manuscript for important intellectual content. All of the authors read and approved the final manuscript.
